# miRdSNP: a database of disease-associated SNPs and microRNA target sites on 3'UTRs of human genes

**DOI:** 10.1186/1471-2164-13-44

**Published:** 2012-01-25

**Authors:** Andrew E Bruno, Li Li, James L Kalabus, Yuzhuo Pan, Aiming Yu, Zihua Hu

**Affiliations:** 1 Center for Computational Research, New York State Center of Excellence in Bioinformatics & Life Sciences, State University of New York at Buffalo, Buffalo NY 14260, USA; 2 Department of Pharmacy, Beijing Jishuitan Hospital, China; 3 Department of Pharmaceutical Sciences, State University of New York at Buffalo, Buffalo NY 14260 USA; 4 Department of Ophthalmology, Department of Biostatistics, Department of Medicine, State University of New York at Buffalo, Buffalo NY 14260 USA; 5 SUNY Eye Institute, Buffalo NY 14260 USA

## Abstract

**Background:**

Single nucleotide polymorphisms (SNPs) can lead to the susceptibility and onset of diseases through their effects on gene expression at the posttranscriptional level. Recent findings indicate that SNPs could create, destroy, or modify the efficiency of miRNA binding to the 3'UTR of a gene, resulting in gene dysregulation. With the rapidly growing number of published disease-associated SNPs (dSNPs), there is a strong need for resources specifically recording dSNPs on the 3'UTRs and their nucleotide distance from miRNA target sites. We present here miRdSNP, a database incorporating three important areas of dSNPs, miRNA target sites, and diseases.

**Description:**

miRdSNP provides a unique database of dSNPs on the 3'UTRs of human genes manually curated from PubMed. The current release includes 786 dSNP-disease associations for 630 unique dSNPs and 204 disease types. miRdSNP annotates genes with experimentally confirmed targeting by miRNAs and indexes miRNA target sites predicted by TargetScan and PicTar as well as potential miRNA target sites newly generated by dSNPs. A robust web interface and search tools are provided for studying the proximity of miRNA binding sites to dSNPs in relation to human diseases. Searches can be dynamically filtered by gene name, miRBase ID, target prediction algorithm, disease, and any nucleotide distance between dSNPs and miRNA target sites. Results can be viewed at the sequence level showing the annotated locations for miRNA target sites and dSNPs on the entire 3'UTR sequences. The integration of dSNPs with the UCSC Genome browser is also supported.

**Conclusion:**

miRdSNP provides a comprehensive data source of dSNPs and robust tools for exploring their distance from miRNA target sites on the 3'UTRs of human genes. miRdSNP enables researchers to further explore the molecular mechanism of gene dysregulation for dSNPs at posttranscriptional level. miRdSNP is freely available on the web at http://mirdsnp.ccr.buffalo.edu.

## Background

Single nucleotide polymorphisms (SNPs) underlie disease susceptibility through their effects on protein function and gene expression. Most identified mutations are non-synonymous SNPs that result in amino acid changes in proteins. It is well known that non-coding disease-associated SNPs (dSNPs) within regulatory regions of the genome can result in gene dysregulation at either transcriptional or posttranscriptional level. One potential source for the latter is SNPs which create, destroy, or modify the efficiency of miRNA binding to the 3'UTR of a gene. Supporting this idea, SNPs within the miRNA target sites of genes have been implicated in hippocampal sclerosis [1], parkinson disease [2], tourette's syndrome [3], asthma [4], cardiovascular disease [5], neurodegenerative disease [6], periodontal diseases [7], tumor susceptibility [8], and various types of cancers [9-12]. Other than SNPs within miRNA target sites, SNPs outside miRNA binding site can affect miRNA function. One recent finding [13] demonstrates that a polymorphism outside the miR-24 binding site in the 3'UTR of human dihydrofolate reductase (DHFR) affects DHFR expression by interfering with miR-24 function, resulting in DHFR over-expression and methotrexate resistance. There is also a report suggesting that SNPs within a certain region on both sides of miRNA target sites have the highest influence on miRNA binding to the target sites and that SNPs on the rest of 3'UTR sequences have impact on miRNA function as well [14].

A few databases have been built to aid researchers in exploring the impact of SNPs on the binding of miRNA and targets. While polymiRTS [15] represents the polymorphism in putative miRNA target sites and their involvement in quantitative trait locus effects, Patrocles database compiles DNA sequence polymorphisms in the 3'UTR of genes in seven vertebrate species that perturb miRNA-mediated gene regulation [16]. The findings of dSNPs on the 3'UTRs have been growing rapidly during the past few years. Furthermore, a few dSNPs [1-5,7-12] have been proven to alter gene expression through modifying specific miRNA target sites, however the molecular mechanism causing diseases for majority of dSNPs on the 3'UTRs is largely not known. There is a strong need to have a database specifically recording dSNPs and tools for capturing their proximity to miRNA target sites on the 3'UTRs so that researchers can explore further the molecular mechanism of gene dysregulation for dSNPs at posttranscriptional level.

Aiming to provide a comprehensive data source of dSNPs affecting posttranscriptional regulation of disease-related genes and tools for exploring the nucleotide distance between miRNA target sites and dSNPs, we present here miRdSNP, a database of manually curated dSNPs on the 3'UTRs of human genes from available publications in PubMed. A robust web interface and advanced search tools are provided showing the nucleotide distance between dSNPs and predicted miRNA target sites from the most popular algorithms, namely TargetScan [17] and PicTar [18]. We also incorporated all SNPs on 3'UTRs of individual genes into the database so the relationship of SNPs with both dSNPs and miRNA binding sites can be analyzed using the web interface. In addition, we also include predicted miRNA target sites generated by dSNPs based on our analysis and annotate genes with experimentally confirmed targeting by miRNAs from four separate curated databases.

## Construction and content

### Data Sources

**Figure 1:**
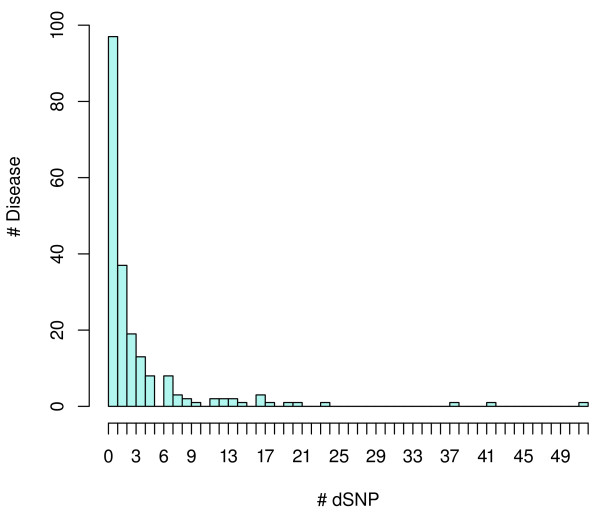
**Histogram of the number of dSNPs associated with individual diseases**.

miRdSNP provides a manually curated dataset of dSNPs on the 3'UTRs of human genes from all PubMed articles linked in Entrez. These include 786 dSNP-disease associations from 630 unique dSNPs and 204 types of diseases. Out of these diseases 97 (47.5%) of them are associated with only 1 dSNP and 153 (75%) are associated with no more than 3 dSNPs (Figure 1). Breast cancer has the highest number with 52 associated dSNPs, followed by type 2 diabetes, schizophrenia, rheumatoid arthritis, obesity, and colorectal cancer with 42, 38, 24, 21 and 20 dSNPs, respectively. The database also incorporates reference sequence (RefSeq) genes, predicted miRNA target sites, and SNP sequence data into a single consolidated resource.

### Database Construction

We obtained 3'UTRs of human RefSeq genes (hg18 March 2006 assembly) from the UCSC Genome browser [19]. A total of 19,834 genes (including introns) were parsed and loaded into miRdSNP and the chromosomal coordinates for each gene were indexed along with the exon lengths. If a gene had multiple transcripts we selected the one with the longest sequence length. We obtained the SNP dataset from UCSC Genome browser (NCBI dbSNP [20] Build 130). Of 18,833,531 SNPs we indexed the genomic coordinates for a subset of 175,351 located on 3'UTRs of 16,810 genes. SNPs aligning to more than 1 locus or mapped to intron regions were excluded. We then annotated dSNPs using an in-house developed data pipeline which searches for PubMed articles linked to SNPs. The data pipeline queries ELink from the Entrez Programming Utilities to find all PubMed IDs linked to SNPs via the "snp pubmed" link. We queried all 3'UTR SNPs and found 2,785 PubMed publications linked to 16,447 unique SNPs. We then manually reviewed these literatures and identified 630 dSNPs for 204 human diseases from 754 publications. The data pipeline for harvesting PubMed-SNP associations from Entrez is automated and the results are displayed in a web interface, allowing multiple users to manually review articles in parallel. This enables us to update the curated dSNP dataset frequently as the literature evolves.

We captured linkage disequilibrium (LD) information for each dSNP using the latest data provided by the HapMap project [21] (version 2009-04_rel27). We downloaded the raw LD files for each population and searched for pairs of genetic variants that included dSNPs. We then indexed all variants in strong LD of each dSNP using an *R*^2 ^≥ 0.80 threshold. Regional LD plots were also generated for each dSNP using a modified version of the R script provided by SNAP [22] and data from the CEU (CEPH Utah Residents with Northern and Western European Ancestry) population.

We obtained miRNA target site datasets from two miRNA target prediction algorithms, namely TargetScan 5.1 and PicTar which were found to have the highest precision and sensitivity out of eight commonly used algorithms [23]. The data for each prediction algorithm was downloaded from the respective source, and the genomic coordinates for each target site were indexed and mapped to RefSeq genes. In addition, each miRNA target site was cross referenced with miRBase [24] and targets referencing dead or non-existent miRNAs were excluded. The genomic coordinates from PicTar were converted from hg17 assembly to hg18 assembly using the LiftOver utility from UCSC. The exon index for each miRNA target site was computed and used to calculate the nucleotide distance between SNPs and predicted miRNA target sites. This distance is calculated from the start or end location of the miRNA target "seed" region (~7nt), depending on whether the SNP is upstream or downstream of the miRNA target site. SNPs which fall inside the miRNA target "seed" region have a distance of 0. To address the low prediction specificity of the miRNA target prediction algorithms we incorporate data from four curation databases (TarBase [25], miRTarBase [26], miRecords [27], and miR2disease [28]) which collect experimentally confirmed miRNA target interactions. Genes with experimentally confirmed targeting by miRNAs were annotated in miRdSNP and displayed with a green check icon within the user interface.

To predict new miRNA target sites created by dSNPs, we first generated candidate sequences using a 6-nt flank up/down stream from each dSNP location replacing the dSNP with the observed allele. Using these new candidate sequences we searched for perfect match 7-mer seed regions from miRBase mature sequence data. We then extracted 25-nt flank upstream of the matching seed region and used the miRNA target prediction program miRanda [29] with default cutoff to further eliminate false positives. We were able to identify 180 newly created miRNA target sites from 138 dSNPs.

Loading and indexing new data in miRdSNP is an automated process allowing for streamlined updates to the database as new RefSeq, miRNA target prediction, and SNP data become available. All data in miRdSNP is available for download in raw text format (CSV, BED) with access to previous versions.

### Utility and discussion

**Figure 2:**
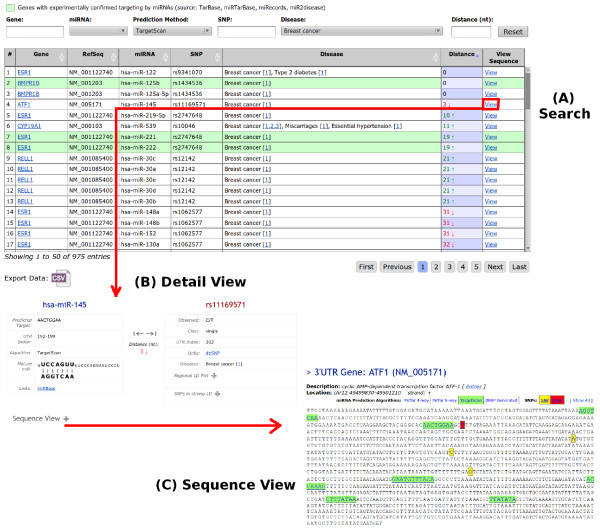
**Overview of miRdSNP web interface**. (A) The search results in tabular format, (B) detail view for miRNA target and dSNP, and (C) sequence view for the selected gene.

miRdSNP provides a publicly accessible web interface for interrogating the database. The advanced search tool as shown in Figure 2A allows the user to perform proximity searches between miRNA targets and dSNPs. Searches can be filtered by gene name, miRBase ID, target prediction algorithm, disease, and any nucleotide distance between SNPs and miRNA target sites. Users can also select to only include search results with experimentally confirmed genes targeted by miRNAs. Search results are displayed in tabular format for browsing and can be exported in plain text (CSV) format for use with external applications. Exporting data is context sensitive within a search enabling users to download only results specific to their area of interest. In addition to dSNPs, users can search over all exon distances between miRNA target sites and SNPs, encompassing over three million records. A detail view of each search result is provided which displays more fine grained information as shown in Figure 2B. Here one can view the SNPs in strong LD and the Regional LD plot for the dSNP of interest. Individual search results can also be viewed at the sequence level showing the annotated locations for miRNA target sites, dSNPs, and SNPs (Figure 2C). Each annotated location on the sequence is click-able, showing detailed information such as mature miRNA sequence, UTR index, and links to miRBase, dbSNP, and PubMed. The sequence views display miRNA target site annotations from multiple prediction algorithms allowing the user to dynamically toggle between them for easy comparison. Along with searching, the miRdSNP web interface provides the ability to browse by gene, displaying all miRNA targets, SNPs, and diseases for every RefSeq gene in the database.

**Figure 3:**
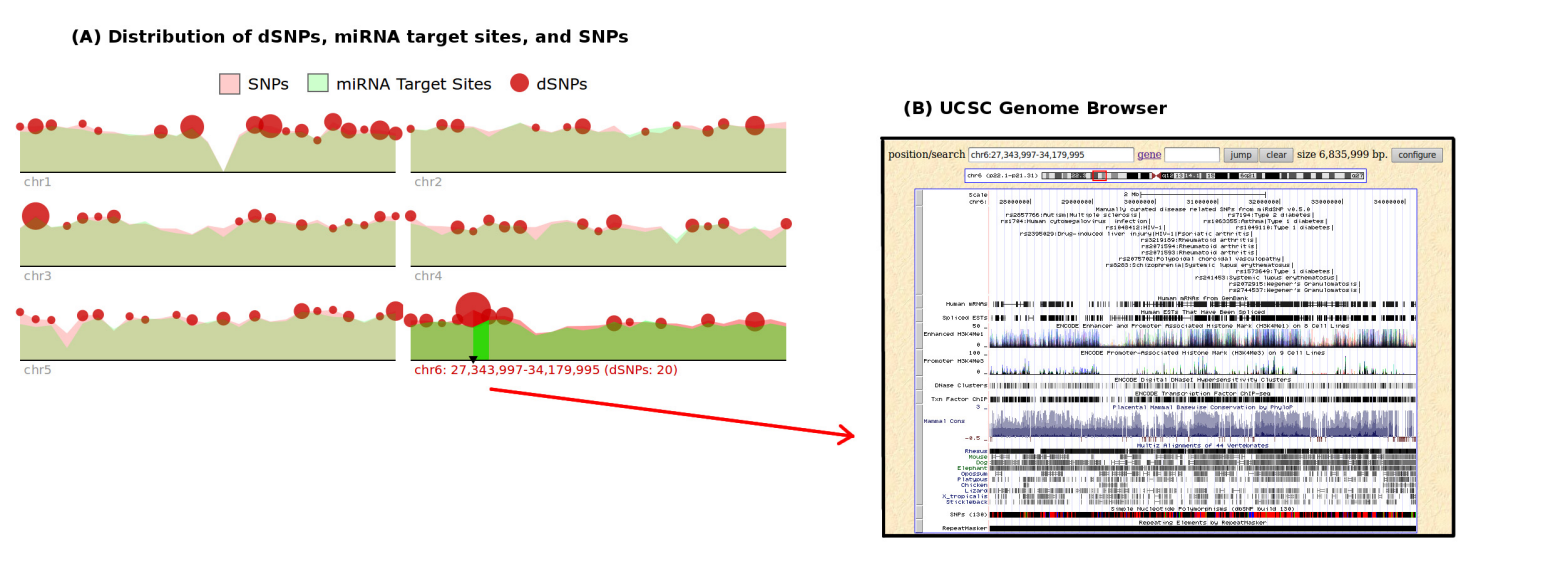
**(A) Distribution of dSNPs, miRNA target sites, and SNPs on individual chromosomes**. The green area curve represents miRNA target site (TargetScan) density, red area curve is SNP density, and red circles represent dSNP density where the larger the radius the more dSNPs in the chromosomal location. (B) Linking dSNPs to the UCSC Genome browser.

An interactive visualization tool is provided for viewing the chromosomal distribution of dSNPs, miRNA target sites (from TargetScan), and SNPs (Figure 3A). Using this tool a user can view the density relationship between miRNA target sites, dSNPs, and SNPs across chromosomes. dSNPs are shown as red circles, the larger the radius the more dSNPs found at that chromosomal location. miRNA target site and SNP densities are displayed as log-normalized area curves. Hovering over a particular region of the chromosome shows the coordinates along with the number of dSNPs in that region. Integration with the UCSC Genome browser is provided using a custom track BED file. Any chromosomal region containing dSNPs is linked directly to the UCSC Genome browser (Figure 3B), allowing the user to view more detailed information for genes around the dSNP location. The interactive tool requires a SVG (Scalable Vector Graphics) compliant browser but we also provide a non-interactive version for users without SVG support.

## Conclusion

miRdSNP is an ongoing effort to create a comprehensive data source for exploring the effect of SNPs on miRNA binding in relation to human diseases. We are working on importing data from other miRNA target prediction algorithms such as DIANA-microT v3.0 [30] and ElMMo [31]. Since the accuracy of the manually curated database of dSNPs is an integral part of miRdSNP, we aim to further broaden the amount of data captured from the manual process. Data such as study design, sample size, and p-values would further enhance the ability to determine the disease-SNP association. We aim to update the dSNP curation database yearly and as new versions of the miRNA target prediction algorithms become available.

## Availability and requirements

miRdSNP is freely available on the web at http://mirdsnp.ccr.buffalo.edu.

## Authors' contributions

ZH conceived of the project. LL, JLK, YP, AY, and ZH participated in the manual curation of disease-associated SNPs. AEB designed and implemented the database, front end web interface, and implemented all data processing. ZH and AEB drafted the manuscript. All authors read and approved the final manuscript.
